# Time to Cross Paths: Neuroplasticity-Informed ACL Rehabilitation that Includes Cross-Education

**DOI:** 10.1186/s40798-025-00967-x

**Published:** 2026-02-09

**Authors:** Tibor Hortobágyi, Dustin R. Grooms, Márk Váczi, Leila Bogdán, Rubén Lara Gómez, Tibor Mintál, Gergely Orsi, Nicola A. Maffiuletti, Justin W. Andrushko, Jonathan P. Farthing

**Affiliations:** 1 Department of Human Movement Sciences, University Medical Center Groningen, University of Groningen, Groningen, The Netherlands; 2 Department of Kinesiology, Hungarian University of Sports Science, Budapest, Hungary; 3 Institute of Sport Sciences and Physical Education, Faculty of Sciences, University of Pécs, Pécs, Hungary; 4 Ohio Musculoskeletal & Neurological Institute (OMNI), Ohio University, Athens, OH USA; 5 Department of Athletic Training, College of Health Sciences and Professions, Ohio University, Athens, OH USA; 6 Department of Physical Therapy, College of Health Sciences and Professions, Ohio University, Athens, OH USA; 7 Departamento de Fisioterapia, Facultad de Ciencias de la Salud, Universidad de Málaga, Málaga, Spain; 8 Grupo de Investigación Clinimetría F14, Instituto de Investigación Biomédica de Málaga y Plataforma en Nanomedicina-IBIMA Plataforma BIONAND, Málaga, Spain; 9 Sports Medicine Center, Medical School, University of Pécs, Pécs, Hungary; 10 Department of Neurology, Medical School, University of Pécs, Pécs, Hungary; 11 Hungarian Research Network, HUN-REN-PTE Clinical Neuroscience MR Research Group, Pécs, Hungary; 12 Human Performance Lab, Schulthess Klinic, Zurich, Switzerland; 13 Department of Sport, Exercise and Rehabilitation, Faculty of Health and Life Sciences, Northumbria University, Newcastle upon Tyne, Tyne and Wear, UK; 14 College of Kinesiology, University of Saskatchewan, Saskatoon, Canada

**Keywords:** Anterior cruciate ligament, Rehabilitation, Cross-education, Neuroplasticity

## Abstract

The efficacy of anterior cruciate ligament (ACL) rehabilitation following reconstruction surgery is sub-optimal and the return-to-sport criteria are inconsistent. We examine the hypothesis that the dysfunctional neuroplasticity induced by an ACL injury could be resolved faster when cross-education is combined with innovative paradigms incorporating visual-cognitive tasks to reduce attentional compensation. We posit that the priming effects could be amplified if therapists combined higher force, eccentric based cross-education exercises with visual-cognitive dual-tasking. The overlapping nature of neuroplasticity after an ACL injury and that induced by cross education may provide a pathway to not only address the mechanical muscle strength deficits associated with injury, but the underlying neurological deficits as well. We provide a practical guide to how neuroplasticity-informed ACL rehabilitation that includes cross-education might accelerate recovery from an ACL injury and the subsequent reconstruction surgery.

## Key Points

- Rehabilitation from the aftermath of an ACL injury and surgery can take up to a year.
- We suggest the inclusion of cross-education and visual-cognitive tasks in ACL rehabilitation.
- Such neuroplasticity-informed ACL rehabilitation could improve long-term outcomes.

## Introduction

Rapid decelerations and change of directions cause ~ 70% of non-contact anterior cruciate ligament (ACL) injuries, requiring reconstructive surgery [1]. Subsequent rehabilitation can last up to a year, with many athletes never able to return to competition and ~ 25% of returners suffering a re-injury [1]. These data suggest that the efficacy of ACL rehabilitation following reconstruction surgery is sub-optimal and the return-to-sport criteria are inconsistent.

## Neuroplasticity After an ACL Injury

In an attempt to maintain function secondary to ACL injury-induced pain, effusion, quadriceps muscle weakness, joint instability, and proprioceptive deficits, the nervous system employs cognitive-motor control compensations [2]. Such compensations increase attentional demand to execute motor actions [2]. The impaired afference and altered sensory integration after injury may cause a dysfunction in the gamma-loop feedback, impair spinal reflex excitability and delay long-latency reflexes, further compounding the reliance on direct attentional based motor control. These changes contribute to quadriceps arthrogenic muscle inhibition, or “muscle activation failure” [2], which necessitates extra neural drive to the motoneurons of muscles surrounding the operated knee [2]. The resulting inhibition creates a feedback cycle of cognitive-motor compensation whereby repeated involvement of frontal brain regions and associated motor planning are needed to meet muscle activation demands once more regulated by spinal or sub-cortical circuits [2, 3].

ACL injury and reconstruction surgery give rise to widespread neuroplasticity that resembles changes in the brain reported after deafferentation induced by limb immobilization, peripheral nerve injury, and amputation [4]. The overall activation profile after ACL reconstruction is likely related to knee pain, neural drive needed for movement planning, compensations for deficits in proprioception and movement execution, increased cognitive load, and a greater reliance on cross-modal processing to move and place the limb accurately. Recently-proposed ACL rehabilitation paradigms are designed based on the dichotomous activation patterns to exploit cross-modal neuroplasticity and visual-cognitive dual-tasking for an accelerated and more efficient treatment [2, 3].

## Cross-Education in ACL Rehabilitation

Researchers have explored a variety of methods to optimize recovery after ACL reconstruction surgery. With a history of ~ 130 years, several sessions of unilateral exercise are known to improve maximal voluntary strength and motor skills in the contralateral, non-practiced homologous muscle pair, a phenomenon termed ‘cross-education’ [5]. Cross-education is a safe, low risk, and effective adjuvant to standard ACL rehabilitation. When exercise of the non-operated limb is added to standard ACL rehabilitation, cross-education seems to improve knee extensor muscle strength of the operated side beyond gains produced by ACL rehabilitation alone [6]. Cross-education produces inter-limb transfer of muscle strength and motor skill through intra- and inter-hemispheric neuroplasticity [5, 7, 8]. Despite encouraging results, inclusion of cross-education in ACL rehabilitation can still be controversial [9, 10].

Reluctance to include cross-education in ACL rehabilitation probably relates to an incomplete mechanistic understanding and a misconception that cross-education increases inter-limb strength asymmetry, even though evidence suggests no detrimental effects of ACL rehabilitation supplemented with cross-education on between-leg symmetry [9]. Importantly, cross-education also offers the potential to support beneficial brain plasticity after ACL reconstruction that can enhance rehabilitation outcomes.

## Neuroplasticity Associated with Cross-Education

Cross-education should be implemented as an adjuvant to standard ACL rehabilitation. Consensus suggests an intervention lasting several weeks, beginning as early as possible following surgery when the operated limb has limited functional capacity [9]. Forceful unilateral resistance training and ankle and knee flexion-extension movements activate distributed networks of brain areas bilaterally that include the primary motor cortex, premotor cortex, and supplementary motor areas [4, 11]. Activation of these areas during cross-education administered soon after surgery could provide a superior neurologic foundation to avoid the maladaptive neuroplasticity typically observed after standard ACL rehabilitation [3]. This is because the unilateral contractions or motor skills performed by the non-operated limb, through bilateral brain activation and inter-hemispheric connectivity, prime the circuits in the hemisphere that controls the operated limb [4, 7, 8]. Therefore, beginning early-phase rehabilitation sessions with cross-education, when the operated limb has limited function, may be an effective neural priming method that can enhance standard rehabilitation protocols performed after exercise of the non-injured limb, which could lead to better long-term outcomes [5, 6, 9, 12, 13].

## Conclusion: Neuroplasticity-Informed ACL Rehabilitation that Includes Cross-Education

Thus, we hypothesize that the dysfunctional neuroplasticity induced by the injury could be resolved faster, especially when cross-education is combined with the recently proposed innovative paradigms incorporating visual-cognitive tasks to reduce attentional compensation. We posit that the priming effects could be amplified if therapists combined higher force, eccentric based cross-education exercises with visual-cognitive dual-tasking [5, 9]. The overlapping nature of neuroplasticity after ACL injury and that induced by cross education may provide a pathway to not only address the mechanical muscle strength deficits associated with injury, but the underlying neurological deficits as well.

Table 1 provides practical examples for novel cross-education exercise to be implemented soon after ACL reconstruction surgery. Such a dual and interactive paradigm shift in cross-education and ACL rehabilitation research would have the potential to facilitate cross-talk between the two schools of researchers who have so far focused solely on cross-education or ACL rehabilitation, respectively. Synchronization between these two areas of research is highly needed in future randomized clinical trials so that the opportunity is not missed to benefit ACL patients by reducing the duration of rehabilitation and the time to return to sport.

**Table 1:** Clinical practice recommendations for adding cross-education to standard rehabilitation during early-phase rehabilitation following ACL reconstruction surgery. Therapists could select from the menu of cross-education programs according to patient and facility characteristics. Therapists could deliver the cross-education program adjuvant to standard care for 10–20 min per session for 8–10 weeks starting soon after reconstruction surgery

Exercise and description	Neuroplasticity and muscle activation to speed early phase ACL rehabilitation combined with cross-education	Clinical tips and neurocognitive add-ons during early phase ACL rehabilitation combined with cross-education
Unilateral seated concentrically-biased knee extension, leg press	Modulates ipsilateral motor cortical and corticospinal and interhemispheric excitability, enhancing cross-education to the injured limb [14]. Cognitive engagement facilitates these processes	Progress from low to high resistance; add neurocognitive tasks (math) or visual target-tracking to engage mental processes. Move in response to ‘Go/No-Go’ cue to engage excitatory and inhibitory mental processes. Vary leg press range of motion, start and stop speed to mimic real-world situations
Unilateral seated eccentrically-biased knee extension	Capitalizes on the unique ipsilateral sensorimotor brain activation associated with eccentric muscle contraction [15]	Use high loads; randomize slow/fast contraction speed; pair movement phase (flexion/extension) with visual stimulus (light flash) or auditory cues to engage cognitive processes and simulate real-world reactions. Combine digital neurocognitive tasks with force production to engage working memory [13]
Prone hamstring curl	Targets knee flexors, stimulating bilateral engagement in brain motor regions to support the injured side’s recovery [16, 17]	Progress from low to high loads; add a memory challenge: recalling words or numbers, for dual-tasking and heightened neurocognitive demand
Single-leg squat with cognitive distraction	Targets knee extensors/knee flexors with a focus on balance, promoting proprioception and motor control; facilitates cross-education benefits for knee stability by modulating ipsilateral motor cortical and corticospinal excitability [18]	Perform single-leg squats aided by light external support; add a visual task (tracking moving objects) for a dual-task scenario mimicking real-world situations

## Data Availability

Not applicable.
